# UNDERSTANDING THE CONNECTION BETWEEN ATTACHMENT TRAUMA AND MATERNAL SELF‐EFFICACY IN DEPRESSED MOTHERS

**DOI:** 10.1002/imhj.21692

**Published:** 2017-12-27

**Authors:** Natalie Brazeau, Samantha Reisz, Deborah Jacobvitz, Carol George

**Affiliations:** ^1^ Mills College; ^2^ Cambridge University and University of Texas at Austin; ^3^ University of Texas at Austin; ^4^ Mills College

**Keywords:** attachment trauma, postpartum depression, maternal self‐efficacy, emotional support, trauma de afectividad, depresión posterior al parto, autoeficacia materna, apoyo emocional, Trauma de l'attachement, dépression postpartum, auto‐efficacité maternelle, soutien émotionnel, Bindungstrauma, postpartale Depression, mütterliche Selbstwirksamkeit, emotionale Unterstützung, 愛着のトラウマ, 産後抑うつ, 母親の自己効力感, 情緒的支援, 依附創傷, 產後抑鬱, 母親自我效能感, 情緒支持, صدمة التعلق, اكتئاب ما بعد ئلولادة, الكفاية الذاتية للأمهات, الدعم العاطفي

## Abstract

Maternal self‐efficacy predicts sensitive and responsive caregiving. Low maternal self‐efficacy is associated with a higher incidence of postpartum depression. Maternal self‐efficacy and postpartum depression can both be buffered by social support. Maternal self‐efficacy and postpartum depression have both been linked independently, albeit in separate studies, to the experience of violent trauma, childhood maltreatment, and spousal abuse. This study proposed a model in which postpartum depression mediates the relation between attachment trauma and maternal self‐efficacy, with emotional support as a moderator. Participants were 278 first‐time mothers of infants under 14 months. Cross‐sectional data were collected online. Mothers completed questionnaires on attachment trauma, maternal self‐efficacy, postpartum depression, and emotional support. A moderated mediation model was tested in a structural equation modeling framework using Mplus’ estimate of indirect effects. Postpartum depression fully mediated the relation between trauma and maternal self‐efficacy. Emotional support moderated only the pathway between postpartum depression and maternal self‐efficacy. Attachment trauma's implications for maternal self‐efficacy should be understood in the context of overall mental health. Mothers at the greatest risk for low maternal self‐efficacy related to attachment trauma also are those suffering from postpartum depression. Emotional support buffered mothers from postpartum depression, though, which has implications for intervention and future research.

## RESUMEN

La autoeficacia materna predice el cuidado sensible y receptivo. Una baja autoeficacia materna se asocia con una más alta incidencia de depresión posterior al parto. La autoeficacia materna y la depresión posterior al parto pueden ambas ser mediadas por el apoyo social. La autoeficacia materna y la depresión posterior al parto han sido ambas relacionadas independientemente, aunque en estudios separados, con la experiencia de trauma violento, maltrato en la niñez y abuso conyugal. Este estudio propone un modelo en el cual la depresión posterior al parto sirve de mediadora con respecto a la relación entre el trauma de afectividad y la autoeficacia materna, con el apoyo emocional como moderador. Las participantes fueron 278 madres primerizas de infantes bajo los 14 meses de edad. Electrónicamente se recogió información con muestras representativas. Las madres respondieron cuestionarios sobre el trauma de afectividad, la autoeficacia materna, la depresión posterior al parto y el apoyo emocional. Se puso a prueba un modelo de mediación moderado dentro del marco de un modelo socio‐ecológico (SEM), usando Mplus para estimar los efectos indirectos. La depresión posterior al parto completamente sirvió de mediadora de la relación entre el trauma y la autoeficacia materna. El apoyo emocional moderó solamente la trayectoria entre la depresión posterior al parto y la autoeficacia materna. Las implicaciones del trauma de afectividad en el caso de la autoeficacia materna deben ser comprendidas en el contexto de la salud mental general. Las madres bajo el mayor riesgo de tener una baja autoeficacia materna relacionada con el trauma de afectividad son también aquellas que sufren de depresión posterior al parto. Sin embargo, el apoyo emocional sirvió para paliar el impacto de la depresión posterior al parto en las madres, lo cual tiene implicaciones para la intervención y la futura investigación.

## RÉSUMÉ

L'auto‐efficacité maternelle prédit les soins sensibles et réactifs. Une auto‐efficacité maternelle basse est liée à une incidence plus élevée de dépression postpartum. L'auto‐efficacité maternelle et la dépression postpartum peuvent toutes deux être amorties par le soutien social. L'auto‐efficacité maternelle et la dépression postpartum ont toutes deux été indépendamment liées, bien que dans des études séparées, à l'expérience d'une trauma violent, à la maltraitance durant l'enfance et aux violences domestiques. Cette étude a proposé un modèle dans lequel la dépression postpartum affecte la relation entre le trauma de l'attachement et l'auto‐efficacité maternelle, avec le soutien émotionnel comme modérateur. Les participantes ont consisté en 278 mères (pour la première fois) de bébés de moins de 14 mois. Des données basées sur une coupe statistique ont été recueillies en ligne. Les mères ont rempli des questionnaires sur le trauma de l'attachement, l'auto‐efficacité maternelle, la dépression postpartum et le soutien émotionnel. Un modèle de médiation modéré a été testé dans une structure SEM en utilisant l'estimation Mplus d'effets indirects. La dépression postpartum a pleinement servi de médiateur à la relation entre le trauma et l'auto‐efficacité maternelle. Le soutien émotionnel n'a modéré que la trajectoire entre la dépression postpartum et l'auto‐efficacité maternelle. Les implications du trauma de l'attachement pour l'auto‐efficacité maternelle devraient être comprises dans le contexte de la santé mentale d'ensemble. Les mères les plus à risque d'auto‐efficacité maternelle basse liée au trauma de l'attachement sont aussi celles souffrant de dépression postpartum. Le soutien émotionnel a servi d'amortisseur à la dépression postpartum pour les mères, cependant, ce qui a des implications pour l'intervention et les recherches futures.

## ZUSAMMENFASSUNG

Die mütterliche Selbstwirksamkeit sagt eine einfühlsame und responsive Fürsorge vorher. Eine geringe mütterliche Selbstwirksamkeit ist mit einer höheren Inzidenz von postpartaler Depression verbunden. Mütterliche Selbstwirksamkeit und postpartale Depression können beide durch soziale Unterstützung gepuffert werden. Sowohl die mütterliche Selbstwirksamkeit als auch die postpartale Depression wurden unabhängig voneinander, wenn auch in getrennten Studien, mit der Erfahrung von Gewalttrauma, Kindesmisshandlung und ehelichem Missbrauch in Verbindung gebracht. Diese Studie schlug ein Modell vor, in dem postpartale Depression die Beziehung zwischen Bindungstrauma und mütterlicher Selbstwirksamkeit mediiert, mit emotionaler Unterstützung als Moderator. Die Teilnehmer waren 278 Erstmütter von Säuglingen unter 14 Monaten. Die Querschnittsdaten wurden online erhoben. Die Mütter füllten Fragebögen zu Bindungstrauma, mütterlicher Selbstwirksamkeit, postpartaler Depression und emotionaler Unterstützung aus. Ein moderiertes Mediationsmodell wurde im Rahmen eines SEM unter Verwendung von Schätzungen der indirekten Effekte mittels Mplus getestet. Die postpartale Depression mediierte die Beziehung zwischen Trauma und mütterlicher Selbstwirksamkeit vollständig. Emotionale Unterstützung moderierte nur den Pfad zwischen postpartaler Depression und mütterlicher Selbstwirksamkeit. Die Auswirkungen von Bindungstrauma auf die mütterliche Selbstwirksamkeit sollten im Kontext der allgemeinen psychischen Gesundheit verstanden werden. Mütter mit dem größten Risiko für eine geringe mütterliche Selbstwirksamkeit im Zusammenhang mit einem Bindungstrauma sind Mütter, die an einer postpartalen Depression leiden. Emotionale Unterstützung diente für die Mütter als Puffer der postpartalen Depression, was mit Implikationen für Interventionen und die zukünftige Forschung einhergeht.

## 抄録

抑うつ状態の母親において 愛着のトラウマと母親の自己効力感との間のつながりを理解する

母親の自己効力感は、感受性があり応答的な育児を予測する。母親の低い自己効力感は、産後抑うつのより高い発症率と関連する。母親の自己効力感と産後抑うつの両者は、社会的支援によって緩衝されることができる。母親の自己効力感と産後抑うつの両者は、別々の研究ではあるが、暴力のトラウマ、子ども時代の虐待、および配偶者からの虐待の経験と、独立して関連していた。この研究は、産後抑うつが、情緒的支援を調整変数として、愛着のトラウマと母親の自己効力感との間の関係を媒介するというモデルを提案する。研究に参加したのは、14か月未満の乳児の初産婦278人だった。横断的データがオンラインで収集された。母親たちは、愛着のトラウマ、母親の自己効力感、産後抑うつ、および情緒的支援についての質問紙を記入した。Mplusの間接効果の推計を用いた共分散構造分析SEMの枠組みで、調整媒介モデルmoderated mediation modelが試された。産後抑うつは、トラウマと母親の自己効力感との間の関係を十分に媒介した。情緒的支援は、産後抑うつと母親の自己効力感との間の経路だけを調整した。母親の自己効力感に対して愛着のトラウマが持つ意味は、精神保健全体のコンテクストで理解されるべきである。愛着のトラウマに関連して母親の低い自己効力感へのリスクがより大きな母親は、産後抑うつを患っている母親でもある。情緒的支援は母親の産後抑うつを緩衝したが、それは介入と今後の研究への意味を持つ。

## 摘要

了解抑鬱症母親依附創傷與母親自我效能的關係

母親自我效能預測敏感和回應性護理。母親自我效能感低與產後抑鬱症發病率高有關。母親的自我效能感和產後抑鬱症都可以通過社會支持來緩衝。在不同的研究中, 母親的自我效能感和產後抑鬱症都獨立的與暴力創傷丶童年虐待和配偶虐待的經歷相關。這項研究提出一個模型, 產後抑鬱症中介依附創傷和母親自我效能的關係, 情緒支持為 **調節變數**。與會者是278名14個月以下嬰兒的第一任母親。線上收集橫斷面數據。母親完成關於依附創傷丶母親自我效能丶產後抑鬱和情緒支持的調查問卷。研究採用Mplus估計間接效應, 在SEM框架下測試中介**調節**模型。產後抑鬱症完全中介創傷和母親自我效能的關係。情緒支持只調節產後抑鬱和母親自我效能的關係。依附創傷對母親自我效能的影響應從整體心理健康的角度來理解。與依附創傷相關自我效能感最低的母親也患有產後抑鬱症。但是, 情感支持緩解了母親產後抑鬱症, 因此這些干預對未來的研究有影響。

## ملخص

فهم الصلة بين الصدمات المرتبطة بالتعلق والكفاية الذاتية الأمومية عند الأمهات المكتئبات

ترتبط الكفاءة الذاتية الأمومية بتقديم الرعاية الحساسة والمستجيبة كما يقترن انخفاض الكفاءة الذاتية بارتفاع معدل اكتئاب ما بعد الولادة. ويمكن للدعم الاجتماعي أن يزيد من الكفاءة الذاتية ويخفض معدل الاكتئاب الأمومي. وقد ربطت دراسات منفصلة بين الكفاءة الذاتية الأمومية واكتئاب ما بعد الولادة وبين التعرض لصدمة عنيفة أو إساءة معاملة في الطفولة أو الاعتداء الزوجي. تقترح الدراسة الحالية نموذجا يمكن فيه لاكتئاب ما بعد الولادة أن يتوسط العلاقة بين الصدمات المرتبطة بالتعلق والكفاءة الذاتية للأمهات بحيث يصبح الدعم العاطفي كمتغير وسيط. اشتملت عينة الدراسة على 728 أم للمرة الأولى وأطفال تحت عمر 14 شهر. وتم جمع بيانات القطاع العرضي عبر الانترنت. واستكمل الأمهات استبيانات عن صدمة التعلق والكفاءة الذاتية للأمهات واكتئاب ما بعد الولادة والدعم العاطفي. وتم اختبار نموذج التوسط في إطار نموذج معادلة الهيكلة (SEM) باستخدام تقديرات المؤثرات الغير مباشرة (Mplus). وقد توسط اكتئاب ما بعد الولادة بشكل كامل العلاقة بين الصدمة النفسية والكفاية الذاتية للأمهات أما الدعم العاطفي فقد توسط فقط المسار بين اكتئاب ما بعد الولادة والكفاءة الذاتية للأمهات. ويجب فهم آثار الصدمة المرتبطة بالتعلق على كفاءة الأمهات في ضوء السياق العام للصحة النفسية. فالأمهات المعرضين لانخفاض الكفاية الذاتية المرتبطة بصدمة التعلق هم أيضا يعانون من اكتئاب ما بعد الولادة. الدعم النفسي العاطفي يمكن أن يجنب الأمهات اكتئاب ما بعد الولادة وهذا يؤكد على أهمية التدخل التجريبي والأبحاث المستقبلية.

**Figure 1:**
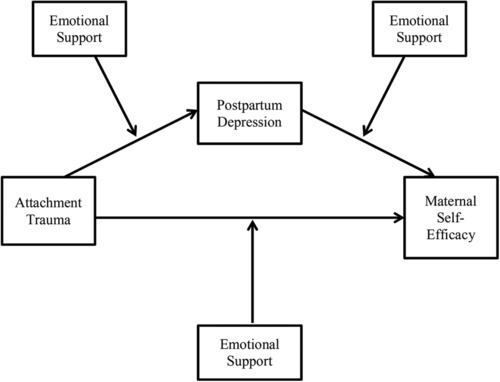
Conceptual model.

The way a woman perceives her ability to parent influences her sense of herself as a mother and the quality of care she provides her child (Teti & Gelfand, 1991). Sensitive and responsive care during the child's first year of life is associated with feelings of maternal self‐efficacy (Coleman & Karraker, 2000, 2003). Thus, it is important to understand why some mothers have a higher sense of self‐efficacy than others. Maternal self‐efficacy is conceptualized as the perception a mother holds regarding her ability to be an effective, reliable caregiver (Bandura, 1982; Jones & Prinz, 2005) and has been examined in relation to the experience of traumatic events, postpartum depression and social support (Cutrona & Troutman, 1986; Kohlhoff & Barnett, 2013; Teti & Gelfand, 1991; Wells‐Parker, Miller, & Topping, 1990). However, the conditions under which trauma disrupts maternal self‐efficacy and the role of postpartum depression and emotional support in diminishing or bolstering a mother's sense of self‐efficacy are not clear. The present study examines attachment trauma—trauma in the context of a close relationship—and the individual's response and evaluation of the event and how that ultimately relates to maternal self‐efficacy. We propose postpartum depression as a mediating factor between attachment trauma and maternal self‐efficacy, and emotional support as a buffer against the effects of attachment trauma (for a conceptual model, see Figure 1).

## LITERATURE REVIEW

Maternal self‐efficacy influences maternal behavior and beliefs, which then impact a mother's parenting practices and overall perception of herself as a competent, effective mother (Coleman & Karraker, 2000, 2003). A mother bases her sense of efficacy on the perception of her ability to understand, plan, and execute actions related to providing care and protection for her child (Leahy‐Warren, McCarthy, & Corcoran, 2009). Bandura (1977) suggested that a number of factors influence maternal self‐efficacy, such as outcome expectancies, social or verbal persuasion, and physiological and mood states. These factors are molded by ongoing experiences and perceptions of failure or success in activities related to the caregiving role.

Maternal self‐efficacy is strongly associated with caregiving behavior (Bandura, 1989; Coleman & Karraker, 1998; Cutrona & Troutman, 1986; Porter & Hsu, 2003). Maternal self‐efficacy can influence women's beliefs about themselves as mothers and contributes to the quality of care a mother provides her child (Coleman & Karraker, 2003). Women with high maternal self‐efficacy are more sensitive, nonpunitive, and attuned to their children than are mothers who do not feel efficacious in the mothering role perhaps because maternal self‐efficacy facilitates positive maternal coping strategies (Unger & Waudersman, 1985; Wells‐Parker et al., 1990).

Maternal caregiving behavior is a strong predictor of child development and outcomes (Donovan & Leavitt, 1985; Lyons‐Ruth & Block, 1996; NICHD Early Child Care Research Network, 2002; Swick & Hassell, 1990; Teti, Gelfand, Messenger, & Isabella, 1995). Parenting self‐efficacy has been positively related to children's cognitive development, compliance, enthusiasm, and affection toward their mother (Coleman & Karaker, 2000, 2003; Donovon & Leavitt, 1989; Swick & Hassell, 1990). Mothers with a compromised sense of efficacy may have a limited ability to meet the needs of their children (Cutrona & Troutman, 1986; Porter & Hsu, 2003). Mothers with lower self‐efficacy are less likely to attempt challenging tasks, such as comforting their crying children, and are more likely to prematurely abandon such a task (Leerkes & Crockenberg, 2002). If mothers routinely fail in their attempts to care for their children, their overall sense of efficacy decreases (Bandura, 1989; Coleman & Karraker, 2003).

High levels of stress and increased incidence of postpartum depression have been linked to low maternal self‐efficacy (Cutrona & Troutman, 1986, Teti & Gelfand, 1991, Wells‐Parker et al., 1990). Mothers with depressive mood states have reported lower levels of self‐efficacy (Cutrona & Troutman, 1986; Donovan, Leavitt, & Walsh, 1990). These mothers also tend to experience anxiety and a lack of control around caregiving tasks (Porter & Hsu, 2003). Indeed, mothers with low maternal self‐efficacy and depression have reported high levels of behavioral problems and child difficulty, and tend to adopt a passive role in parenting (Coleman & Karraker, 2003; Wells‐Parker et al., 1990). The combination of these factors impedes mothers’ caregiving efforts and abilities, resulting in compromised caregiving. Thus, the major goal of this study was to identify why some mothers have higher maternal self‐efficacy than do others.

Maternal self‐efficacy is largely rooted in a person's own childhood experiences (Alvarez‐Segura et al., 2014). Mothers’ negative childhood experiences can threaten the integrity of their relationship with their children (Lyons‐Ruth & Block, 1996). It has been established that women who have been exposed to trauma are at an increased risk of lower maternal self‐efficacy (Caldwell, Shaver, Li, & Minzenberg, 2011; Leerkes & Crockenberg, 2002). However, to our knowledge, no study has examined attachment‐specific trauma and how it influences mothers’ sense of efficacy. Attachment trauma is defined as *all* experiences that threaten the attachment relationship or threaten the integrity of self (George & West, 2012). Attachment trauma is often thought as occurring in childhood; however, these experiences also can occur later on in adulthood and with adult attachment figures (e.g., intimate partner violence). Further, the threat of losing attachment relationships can be similarly traumatic in adulthood as in childhood, such as fear that one's partner could die.

Childhood experiences that threaten the child–parent attachment relationship may hinder mothers’ ability to detect and respond to their children's need for care (Bowlby, 1969/1982, 1980; George & Solomon, 2008; Solomon & George, 2011). If women felt loved and cared for in childhood, they are likely to provide those same feelings and experiences for their own children. However, if they experienced attachment‐related adverse childhood experiences such as neglect, parental loss, or overprotectiveness, these experiences may influence their perception of themself as a worthy and capable parent (Lyons‐Ruth & Block, 1996; Kohlhoff & Barnett, 2013).

The impact of a traumatic event can vary depending on how individuals interpret and respond to that event (Herman, 1997). Thus, it follows that certain types of trauma can be more detrimental to some people than they are to others. Studies examining trauma typically have assessed whether an event has occurred, not the person's reaction to it. The current study assessed attachment trauma specifically as it relates to feelings of distress and helplessness that are reported as associated with trauma. Some studies have found relational trauma to be associated with postpartum depression (Koenen & Widom, 2009; Mezey, Bacchus, Bewley, & White, 2005), but we are unaware of any study to date that specifically has found associations between experiences of attachment trauma and postpartum depression.

Attachment trauma can influence a mother's sensitivity to her child as well as her mental representation of herself as a mother (Lyons‐Ruth & Block, 1996). Bowlby (1969/1982, 1988) conceived of this process as the “internal working model,” which is a cognitive‐emotional framework of a person's mental representation of the self, others, and the world. This internal model interprets, regulates, and predicts attachment‐related behavior. In addition, the internal working model is thought to be the foundation of individuals’ expectations regarding the availability and responsiveness of loved ones in intimate relationships (i.e., attachment figures) (Bowlby, 1988; Bretherton & Munholland, 2008). Self‐efficacy and the internal working model are interconnected—women's life experiences and perceptions comprise their internal working models and influence their perceptions of caregiving and parenting. Representational models are conceived as organizing the way women evaluate their competency as mothers, impacting their ability to tend to their infants’ needs and cues (George & Solomon, 2008, 2011). Childhood experiences strongly influence mothers’ internal working models of caregiving (Bowlby, 1969/1982; George & Solomon, 2008). Women, who experienced abuse in childhood by a primary caregiver, or the failure of this caregiver to protect them from the abuse of other family members, may have compromised internal working models, which can undermine their ability to provide security for their children (Leon, Jacobvitz, & Hazen, 2004; Solomon & George, 2011).

The experience of attachment trauma can compromise a mother's ability to adequately care for her child (Solomon & George, 2011). For women with trauma histories, the presence of a baby may evoke memories of abuse and feelings of depression related to becoming a caregiver (Schore, 2001; Solomon & George, 2008). Women who have experienced attachment trauma may interpret and perceive innocuous events as harmful or scary, which may affect overall functioning and feelings of self‐efficacy (Leerkes & Crockenberg, 2002). Putnam (2003) found that a large proportion of mothers with a history of trauma reported fearing for their child's safety and protection significantly more than did mothers without a trauma history. Women with histories of abuse are often concerned with their ability to protect their children, thus impacting their overall perception of self‐efficacy (Alvarez‐Segura et al., 2014). Putnam conjectured that fear may be linked to feelings of helplessness, lowered self‐efficacy, and depression. These findings suggest that trauma history can result in compromised caregiving.

Trauma also negatively impacts mental well‐being in part as a result of the distress that it causes, which then interferes with the person's ability to function (Davidson, Shannon, Mulholland, & Campbell, 2009; Murphy et al., 2014). Individuals’ response to the trauma may be what impedes their mental functioning rather than the trauma itself—an interpretation that is consistent with attachment theory (Bowlby, 1980). Being the victim of trauma often creates feelings of helplessness as a result of a person's inability to achieve protection, either by not being able to protect him‐ or herself or by not receiving protection from another such as the primary caregiver, also referred to as the “attachment figure.” Feelings of helplessness as a result of not being protected are often heightened when the source of the trauma is an attachment figure (Becker‐Stoll, Fremmer‐Bombik, Wartner, Zimmerman, & Grossmann, 2008; Herman, 1997). Traumatic experiences with attachment figures can negatively impact women's representations of themselves in the caregiving role (Bowlby, 1969, 1973; Slade, Cohen, Sadler, & Miller, 2009; Solomon & George, 2011). Maternal self‐efficacy is rooted in this internal representation. Therefore, it is likely that maternal self‐efficacy is related to women's subjective experiences of trauma, especially when the trauma occurred with an attachment figure.

Direct relations between trauma and maternal self‐efficacy are not well‐understood because most studies have focused on the fact that the traumatic event occurred rather than assessing an individual's response to trauma. Women who have been exposed to trauma tend to experience depression after the birth of their children (Beydoun, Al‐Sahab, Beydoun, & Tamim, 2010; Leeners, Richter‐Appelt, Imthurn, & Rath, 2006; Plaza et al., 2012; Seng et al., 2013). These studies have found that the traumatic events that contribute to postpartum depression tend to be relational, involving someone who was close to the person (Koenen & Widom, 2009; Mezey et al., 2005). Postpartum depression, in turn, has been associated with maternal self‐efficacy (Leerkes & Crockenberg, 2002; Teti & Gelfand, 1991). Mothers with postpartum depression tend to appraise their parenting more negatively than do mothers without depressive symptoms (Leahy‐Warren et al., 2009; Kohlhoff & Barnett, 2013). If a traumatic event has a negative impact on mothers’ well‐being, it follows that this compromised emotional state would influence her sense of efficacy as a mother.

The combination of trauma and postpartum depression is thought to compromise caregiving to an even greater extent than either of these experiences alone (Leeners et al., 2006; Plaza et al., 2012). Women who have experienced trauma or depression may perceive themselves as less capable of providing for their children and may consequently have a lowered sense of self‐efficacy (Leahy‐Warren et al., 2009). Women with trauma histories and postpartum depression reported more difficulties with their children and lower levels of self‐efficacy than did mothers who were not depressed and trauma‐exposed (Cutrona & Troutman, 1986; Porter & Hsu, 2003; Teti & Gelfand, 1991). It is a difficult cycle to break. When mothers’ perceive their parenting as inadequate, past trauma may resurface, which could increase depressive symptoms, making perceptions of their own parenting even more negative (Cutrona & Troutman, 1986; Leahy‐Warren et al., 2009). Given that trauma is significantly related to postpartum depression, it is possible that the degree to which trauma impacts maternal self‐efficacy is mediated, at least partially, by postpartum depression.

It is important to understand the conditions under which postpartum depression negatively affects caregiving because the incidence of postpartum depression is high. According to the American Psychological Association (2016), postpartum depression affects one in seven women in the general population and is considered the most common cause of maternal morbidity in the United States (Robertson, Grace, Wallington, & Stewart, 2004). Women's hormonal, physical, and psychological shifts create increased vulnerability across pregnancy and the postpartum period (Egliston & Rapee, 2007; Heim, Newport, Mietzko, Miller, & Nemeroff, 2008; Slade et al., 2009). The intensity of these changes may be exacerbated in women with a trauma history, potentially increasing depressive symptomology or eliciting the onset of postpartum depression.

Attachment trauma occurs in the context of relationships and has negative implications for postpartum depression and maternal self‐efficacy (e.g., Leahy‐Warren et al., 2009; Plaza et al., 2012). Following this, it is possible that positive experiences in relationships could provide a buffer against these risks. Social support is defined as interpersonal interactions that provide emotional assistance, stress reduction, and esteem (Leahy‐Warren et al., 2009). The subjective experience of feeling supported has been shown to be an important component of social support (Morelli, Lee, Arnn, Zaki, & Morelli, 2015). Social support includes both the perception of available support and the satisfaction of the received assistance (Leerkes & Crockenberg, 2002).

Social support facilitates the maintenance of self‐esteem, therefore contributing to a woman's adaptive coping abilities (Cutrona, 1984; Cutrona & Troutman, 1986). These adaptive skills are essential for new mothers, especially those with a trauma history and/or postpartum depression. Research has found that partner support and parent support are more predictive of maternal self‐efficacy and impact mother–infant interaction more prominently than do other forms of support (Leerkes & Crockenberg, 2002; Longfellow, Zelkowitz, Saunders, & Belle, 1979). Social support protects against physical and mental illness (Balaji et al., 2007; Berkman, Glass, Brissette, & Seeman, 2000). A range of mental health outcomes have been related to the availability of social support networks, including anxiety, depression, stress, and psychological well‐being (Barnett & Gotlib, 1988; Kawachi & Berkman, 2001). A recent study by Ammerman et al. (2013) examined the mediational role of social support in mothers with trauma histories. This study included 208 first‐time mothers who self‐identified as having childhood trauma histories. The participants were recruited through a home‐visiting program and were assessed at 5 months’ postpartum. The Interpersonal Support Evaluation List (ISEL; Cohen & Hoberman, 1983) was used to assess for available tangible and emotional social support. They found that social support mediated mothers’ parenting stress and trauma symptoms when the babies were 5 months old. This underlines the importance of social support for mothers, especially when there is a history of trauma.

The current study focuses specifically on the role of emotional support and how feeling supported and valued within close relationships can influence a mother's depressive symptomology, trauma experiences, and efficacy. It is posited that emotional support can buffer maternal self‐efficacy because when women feel emotionally supported and valued in their relationships, their overall self‐efficacy will likely increase (Marcus, Flynn, Blow, & Barry, 2003; Mezey et al., 2005). We adapted the Interpersonal Support Evaluation: Short Form (ISEL‐12; Cohen, Mermelstein, Kamarck, & Hoberman, 1985) to measure women's feelings of being emotionally supported. This approach aligns with the assessment of the subjective experience in which attachment trauma is measured. To understand the maternal experience of compromised caregivers, it is essential to assess women's subjective experiences of motherhood rather than of both the traumatic event and their perception of motherhood and all it entails.

This is the first study to examine the combined effects of attachment trauma, postpartum depression, and emotional support as they relate to maternal self‐efficacy. Specifically, this study proposed a model in which postpartum depression mediates the relation between attachment trauma and maternal self‐efficacy; emotional support was proposed as a moderator for the pathways between attachment trauma, postpartum depression, and maternal self‐efficacy (for a conceptual model, see Figure 1). Our hypotheses are listed next.

**H1**.: The subjective evaluation of attachment trauma will be associated with maternal self‐efficacy and postpartum depression.
**H2**.: Postpartum depression will be directly related to maternal self‐efficacy.
**H3**.: Postpartum depression will mediate the relation between relationship trauma and maternal self‐efficacy.
**H4**.: Emotional support will moderate the pathways between relationship trauma, postpartum depression, and maternal self‐efficacy.

## METHOD

### Participants

The 280 mothers in this study self‐identified as currently experiencing or having previously experienced postpartum depression and were actively seeking treatment or support through local mental health services or online support groups. Infant age ranged from 1 to 14 months (*M* = 8.23). Mother ages ranged from 19 to 49 years (*M* = 30.39). Participants were predominantly married or cohabiting (92.9%, *n* = 260) and had a mode household income of $75,000 (37.5%, *n* = 105). The majority of mothers identified as Caucasian (87.1%, *n* = 244), 4.3% were Hispanic (*n* = 12), 0.7% were African American (*n* = 2), and 7.9% identified their ethnic background as “other” (*n* = 22). Although 436 participants began the online survey, only the 280 who completed all the measures were included in the present study. Chi‐square tests and *t* tests did not reveal any significant demographic differences between mothers who did and did not fully complete the survey. Additional descriptive information is located in Table 1.

**Table 1:** Descriptive Frequencies

		Frequency	%
Household Income	<$5,000	15	5.4
	$5,000–10,000	10	3.6
	$10,000–25,000	25	8.9
	$25,000–50,000	71	25.4
	$50,000–75,000	54	19.3
	>$75,000	105	37.5
Ethnicity	African American	2	0.7
	Hispanic	12	4.3
	Other	22	7.9
	Caucasian	244	87.1
Marital Status	Married/Cohabiting	260	92.9
	Dating at Least 3 Months	3	1.1
	Divorced/Separated	13	4.6
	Other	2	0.7
Employment Status	Employed	145	51.8
	Unemployed	135	48.2

### Procedure

Mothers were recruited online and in person through parenting magazines, online postpartum support groups, community boards, local online parenting networks and blog groups, and by word of mouth. The study advertisement invited mothers who were experiencing postpartum depression to participate in an online study. The first page of the survey was an informed consent form that notified participants about the general purpose of the study and emphasized their ability to withdraw from the study at any time without consequences. The survey included a demographic questionnaire, the Edinburgh Postnatal Depression Scale (EPDS; Cox, Holden, & Sagovsky, 1987), the Attachment Trauma Life Events Questionnaire: Short Form (Keeling & George, 2013), the Maternal Self‐Efficacy Scale (MSES; Teti & Gelfand, 1991), and the ISEL: Shorted Version (Cohen et al., 1985).

### Measures

#### Experience of attachment trauma

The Attachment Trauma Life Events Questionnaire: Short Form (Keeling & George, 2013) was used to assess experiences of distress and/or helplessness as a result of attachment trauma. This questionnaire was designed to represent a comprehensive list of life events defined as experiences associated with risk for attachment trauma. The concept for the development of this questionnaire was to create a measure with the purpose of assessing an individual's experiences of attachment trauma, defined by George and West (2012) as experiences that threaten to rupture the protective attachment–caregiving relationship or threaten the integrity of self. This approach uses a conceptually derived model of attachment trauma and extends typical attachment‐research approaches using a life‐events model. According to attachment theory, events defined as traumatic can be so threatening and frightening that blocked access to attachment figures and feelings of helplessness can undermine regulatory processes and place an individual at risk for chronic representational, behavioral, and physiological dysregulation and helplessness to protect the self and others (Solomon & George, 2011).

The questionnaire asks participants whether they have experienced a particular event and to rate how distressed/helpless they felt as a result on a 6‐point Likert scale (0 = *I did not experience this*, 1 = *not distressed/helpless*, 3 = *moderately distressed/helpless*, 5 = *very distressed/helpless*). Example items include: “My parent died,” “I was separated from my parent for more than a month as a child,” and “My partner gets very gloomy, depressed, won't speak to me for several days.” The scores are averaged so that each participant's final score represented the average of their experience of distress/helplessness in the face of attachment trauma over the course of their life.

The Attachment Trauma Life Events Questionnaire: Short Form (*blind*) has been related to theoretically derived hypotheses. Keeling (2013) found a significant positive correlation between maternal childhood trauma and child abuse risk in a sample of 86 mothers with children between the ages of 1 and 3 years. Newton, Laible, Carlo, Steele, & McGinley (2014) found a significant negative association between adult total trauma and adult attachment representational integration (i.e., security).

#### Maternal self‐efficacy

Maternal self‐efficacy was assessed using the MSES (Teti & Gelfand, 1991). The MSES contains 10 items rated on a 4‐point Likert scale. Nine questions ask mothers to assess how good they are with their baby in different situations (e.g., “How good are you at getting your baby to pay attention to you?”). The 10th question is a global self‐assessment that asks participants “In general, how good a mother do you feel you are with your baby?”). This tool has demonstrated validity and reliability across numerous studies and is often used in studies of the development of the mother–infant relationship (e.g., Fulton, Mastergeorge, Steele, & Hansen, 2012; Hess, Teti, & Hussey‐Gardner, 2004; Huth‐Bocks, Levendosky, Bogat, & von Eye, 2004; Troutman et al., 2012).

#### Postpartum depression

The EPDS (Cox et al., 1987) was used to assess postpartum depressive symptomology. The EPDS is a self‐report questionnaire that is used to screen for the risk of developing postpartum depression. Respondents are asked to answer questions about their feelings over the previous 7 days (e.g., “I have been able to laugh and see the funny side of things.”). The EPDS has 10 items that are rated on a Likert scale ranging from 0, for “No, not at all,” to 3, for “Yes, most of the time,” with a maximum score of 30. The suggested clinical cutoff is 13 (Matthey, Hensaw, Elliott, & Barnett, 2006). The EPDS has demonstrated consistently high reliability and validity across several cultures and countries for detection of postpartum depression (e.g., Bunevicius, Kusminskas, & Bunevicius, 2009; Gibson, McKenzie‐McHarg, Shakespeare, Price, & Gray, 2009). Internal consistency for the present study was adequate, α = 0.69.

#### Emotional support

Emotional support was assessed using the ISEL‐12 (Cohen et al., 1985). The ISEL consists of 12 items that are rated on a Likert scale of 0 to 3, which are summed to create an overall score. The ISEL‐12 includes three subscales: Appraisal Support, Belonging Support, and Tangible Support. The ISEL‐12 is a widely used tool that has demonstrated validity and reliability with samples of depressed and/or traumatized women in previous studies (e.g., Constantino, Sekula, Rabin, & Stone, 2000; Crane & Constantino, 2003; Dobkin et al., 2007).

This study was specifically concerned with attachment‐related events and appraisals. To assess women's access to support that is more theoretically related to attachment, we identified items that were specific to emotional support and emotional closeness to other people (e.g., “I feel that there is no one I can share my most private worries and fears with”). Internal consistency was calculated for the newly created scale, which included four items with α = .833. This was then used as an independent scale to assess emotional support.

## RESULTS

### Analytic Approach

**Figure 2:**
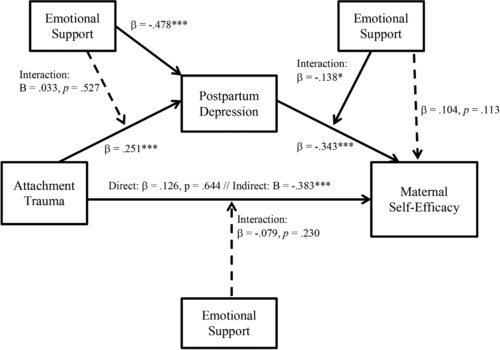
Final model.

A structural equation modeling framework was employed using Mplus Version 7 (Muthén & Muthén, 1998–2012). Path analysis was used for simultaneous testing of both direct and mediating effects, which an ordinary least squares (OLS) regression approach does not allow (Stage, Carter, & Norma, 2004). Indirect effects were assessed using the Mplus estimation of indirect effects, which uses delta method *SE*s to estimate indirect effects, and Sobel's (1982) asymptotic *z* test. Moderation was assessed using interaction terms that were created with the centered variables. The final model can be seen in Figure 2.

### Preliminary Analyses

Descriptive information is reported in Table 1. Pearson's *r* correlations were used to determine the relation among the demographics and the variables of interest (see Table 2). Any demographic variables that significantly covaried with the variables of interest were included in the models as control variables. Maternal depression was inversely correlated with age and positively correlated with income. Higher income mothers reported more depressive symptomology, but also reported more emotional support. In addition, maternal self‐efficacy was correlated with infant age.

**Table 2:** Correlations Between Variables of Interest

	Maternal Age	Baby Age	Household Income	Ethnicity	Attachment Trauma	Postpartum Depression	Maternal Self‐Efficacy	Emotional Support
Maternal Age	1							
Baby Age	.06	1						
Household Income	.34**	−.04	1					
Ethnicity	−.003	.04	−.02	1				
Attachment Trauma	−.02	.08	.02	.01	1			
Postpartum Depression	−.17**	.01	−.19**	−.03	−.06	1		
Maternal Self‐Efficacy	.06	.12*	−.001	.003	.11	−.40**	1	
Emotional Support	−.07	−.06	.23**	.10	−.03	−.54**	−.28**	1

*p < .05. **p < .01.

This study recruited for mothers who self‐identified as having experienced postpartum depression; thus, we checked whether the distribution was skewed with higher numbers of participants scoring higher on postpartum depression. The EPDS distribution was relatively normal and slightly kurtotic (skewness = .242, kurtosis =  −.779). The mean in this sample was 12.59, with scores ranging from 2 to 27 of a possible score of 30. The recommended clinical cutoff for the EPDS is 13 (e.g., Matthey, Henshaw, Elliott, & Barnett, 2006), and 53.6% of this sample scored at or below 12. This highlights the fact that women who report experiencing postpartum depression may only score on subclinical levels. This sample distribution allowed for a more comprehensive examination of how these processes might work for women across the spectrum of postpartum depressive symptoms.

Every form of relationship trauma assessed was experienced by at least 4 people in this sample. The most common relationship trauma was “afraid that my partner will die or be killed,” which was reported by 167 of the 280 participants. Of these individuals, 27 (16.17%) of them reported this as a 5 of 5 for resulting distress and helplessness. The most distressing trauma was the death of a parent before the age of 17. Eleven people experienced this; 7 of those (63.63%) rated it as 5 of 5 for resulting distress and helplessness. The most common traumas are reported in Table 3, and the most distressing traumas are reported in Table 4.

**Table 3:** Five Most Common Attachment Trauma Experiences for Each Category (*N* = 280)

Attachment Trauma	Item	No. Experienced
Parent Trauma (<17)	My parent is very gloomy, depressed, or won't speak to me for several days. (<17)	107
	My parent has a psychiatric disorder or mental illness. (<17)	109
	My parent uses alcohol or drugs in a way that is not good for them. (<17)	100
	My parent is inclined to tease, ridicule, humiliate, or intimidate me to make me feel small. (<17)	126
	My parent becomes unpredictable, angry, or flies out of control. (<17)	140
Parent Trauma (>18)	I am afraid that my parent will die or be killed. (>18)	140
	My parent is very gloomy, depressed, or won't speak to me for several days. (>18)	115
	My parent has a psychiatric disorder or mental illness. (>18)	128
	My parent is inclined to tease, ridicule, humiliate, or intimidate me to make me feel small. (>18)	111
	My parent becomes unpredictable, angry, or flies out of control. (>18)	116
Partner Trauma	I am afraid that my partner will die or be killed.	167
	My partner gets very gloomy, depressed, or won't speak to me for several days.	80
	My partner uses alcohol or drugs in a way that is not good for him or her.	58
	My partner is inclined to tease, ridicule, humiliate, or intimidate me to make me feel small.	63
	My partner can become unpredictably enraged or flies out of control.	75

**Table 4:** Five Most Distressing Attachment Trauma Experiences for Each Category, Rank Ordered

Attachment Trauma	Item
Parent Trauma (<17)	My parent died (<17)
	My parent injures or assaults his or her partner (< 17)
	My parent threatens or has tried to kill him‐ or herself (<17)
	My parent sexually interferes with me or expects me to touch their genitals (<17)
	My parent becomes unpredictable, angry, or flies out of control (<17)
Parent Trauma (>18)	My parent died. (>18)
	My parent threatens or has tried to kill him‐ or herself. (>18)
	My parent uses alcohol or drugs in a way that is not good for them. (>18)
	My parent sexually interferes with me or expects me to touch their genitals. (>18)
	My parent becomes unpredictable, angry, or flies out of control. (>18)
Partner Trauma	My partner died.
	My partner physically punishes me more than most couples, or beats, injures, or assaults me.
	My partner gets me to do what they want by threatening to leave or abandon me.
	My partner threatens or has tried to kill him‐ or herself.
	My partner sexually forces him/herself on me (e.g., unwanted sex, rape).

### Direct Effects

The first research question asked whether the subjective experience of attachment trauma related to maternal self‐efficacy. An OLS regression showed that trauma had a direct effect to maternal self‐efficacy, β = −.13, *p* < .05. The second research question asked whether the subjective experience of attachment trauma related to postpartum depressive symptomology; that is, would women who experienced higher levels of distress and/or helplessness as a result of attachment trauma have a higher likelihood of experiencing more intense postpartum depressive symptomology? There were direct effects from attachment trauma to postpartum depression, β = .251, *p* < .001.

### Indirect Effects

The third research question asked whether the postpartum depression mediated the relation between attachment trauma and maternal self‐efficacy. The mediation model fit the data well, χ2(4) = 4.36, root mean square error of approximation = .018, Comparative Fit Index = .99. In addition, postpartum depression fully mediated the relation between attachment trauma and maternal self‐efficacy, indirect effects estimate: β = −.15, *p* < .001. This indicates that the direct relation between attachment trauma and maternal self‐efficacy was no longer significant once postpartum depression was included in the model.

### Moderation Effects

The fourth research question addressed the role of emotional support in the context of this model. Emotional support had a direct effect to postpartum depression, with lower emotional support relating to more severe depressive symptomology, β = −.478, *p* < .001. Emotional support did not have a direct effect on maternal self‐efficacy. Emotional support was examined as a continuous moderator. It was hypothesized that emotional support would moderate both the pathways between trauma and postpartum depression and between postpartum depression and maternal self‐efficacy. Emotional support did not moderate the pathway from trauma to postpartum depression, but it did moderate the pathway between postpartum depression and maternal self‐efficacy, β = −.138, *p* < .05.

### Alternate Models

To determine whether emotional support played a more important role over social support generally, the model was run with the original ISEL subscales, and the overall social support score. None of those models fit the data well. This indicates that emotional support plays a unique moderating role in the relation between postpartum depression and maternal self‐efficacy.

The moderated mediation model was examined separately for attachment trauma with their parent(s) and for attachment trauma with their partner. The model fit indices for both models were identical to the fit indices for the moderation model that used the combined trauma items. There also were no differences in the estimates of indirect effects.

## DISCUSSION

Maternal self‐efficacy is a strong predictor of mothers’ caregiving behavior. Yet, the reasons that mothers do not feel efficacious as parents are not well‐understood. This study sought to delineate pathways between the experience of attachment trauma and mothers’ sense of efficacy. Trauma in general has been shown to hinder a mother's sense of self‐confidence (e.g., Caldwell et al., 2011; Leerkes & Crockenberg, 2002). This study provides data demonstrating that distress stemming from traumatic experiences with attachment figures (attachment trauma) specifically is problematic for mothers’ confidence in their ability to adequately parent their children. Moreover, this study found that the way attachment trauma affects maternal self‐efficacy is through its larger impact on a mother's mental health. As predicted, postpartum depression fully mediated the relation between a distress/helplessness response to attachment trauma and maternal self‐efficacy. Further, emotional support moderated the pathway between postpartum depression and maternal self‐efficacy. This distinct mediating pathway of depression coupled with the buffering role of emotional support reveals a point of entry for intervention to prevent compromised caregiving in mothers with a trauma history.

Findings from this study highlight the role of postpartum depression in the relation between experiences of distress and helplessness stemming from attachment trauma and maternal self‐efficacy. Postpartum depression fully mediated the pathway between attachment trauma and maternal self‐efficacy. A woman who has experienced attachment trauma or postpartum depression may be at a disadvantage when it comes to her ability to consistently and sensitively tend to the needs of her infant (Schore, 2001; George & Solomon, 2008). This study's findings have helped to elucidate the mechanism by which attachment trauma may come to impair mothers’ views of themselves as competent caregivers. Longitudinal research that tracks social support and mental health across the transition to parenthood for women who have been exposed to trauma is the next step in clarifying these pathways.

This study found that higher levels of emotional support were related to lower levels of postpartum depression. This finding is consistent with that of previous work which has found that higher levels of social support are associated with better mental health and lower levels of depressive symptomatology (Levy‐Shiff, Dimitrovsky, Shulman, & Har‐Even, 1998; Terry, Rawle, & Callan, 1995). Social support can play a protective role in the postpartum period by reducing the stress associated with the transition to motherhood (Collins, Dunkel‐Schetter, Lobel, & Scrimshaw, 1993). For the purposes of this study, we narrowed the scope of social support to specifically examine emotional support in the context of motherhood. Emotional support research provides unique insight into mothers’ perceptions of belonging and well‐being, and thus merits being empirically explored as distinct from other types of support (e.g., instrumental, tangible; Morelli et al., 2015).

The level of emotional support that mothers’ received influenced the degree to which postpartum depression affected their self‐efficacy as a parent. This finding suggests that the women who are able to connect with others enough to receive emotional support are less likely to experience postpartum depression, even if they have experienced highly distressing attachment trauma. This has implications for interventions with depressed mothers who are coping with the challenges of caring for a baby. If they can Finding someone who can provide adequate emotional support can provide a buffer for potential declines in maternal self‐efficacy (Collins et al., 1993). Taken together, this points toward emotional support as uniquely helpful to mothers with trauma histories and/or experiencing postpartum depression. However, the relation between attachment trauma and postpartum depression did not depend on the level of emotional support that mothers received. Attachment trauma can be characterized by a distinct lack of support (e.g., neglect) or being hurt by someone close to you (e.g., abuse by a parent), both of which could make it difficult to trust someone enough to accept emotional support from that person. Thus, attachment trauma may impact one's ability to connect with and receive emotional support.

One limitation of the study is that the mothers in this study were largely recruited from online support groups for depressed mothers. It also may have biased the sample toward women who have access to the Internet, use the Internet more than do others, are more likely to seek support online than in‐person support, and/or may be more socially isolated. Online support groups can be convenient for mothers who are busy and would not be able to attend a group in person but can do so from a computer at some point during the day. It also is possible that it is more likely to draw women who are more socially isolated to begin with—such women who may not have social relationships that they feel they can draw on in person. However, recruiting mothers from online groups allowed us to oversample mothers experiencing postpartum depressive symptomatology and thus to examine pathways related to postpartum depression in more depth, which better served the goals of the study.

Future research should examine different types of attachment trauma (e.g., threats to physical safety, emotional abuse, emotional neglect, or death of a parent) with a wider range of maternal mental health outcomes; this would provide further insight into the complexities of attachment trauma and maternal mental health. Assessing mothers’ attachment representation as they relate to their trauma also would offer another route by which to explore the effects of attachment trauma. By closely examining the pathways between attachment representations and the original traumatic event, we would gain insight into the long‐lasting effects of attachment trauma. Furthermore, to gather more information about trauma and its effects on postpartum depression and maternal self‐efficacy, it is advised that future studies inquire whether mothers have ever received formal therapy for trauma and depression. This information would help to further contextualize the sample's trauma and mental health. It also may be useful to inquire about the length and details of the treatment, including whether they thought it was effective, and if so, what was most beneficial. This could help to uncover whether the relationship to the person providing support and the kinds of support will be most effective in reducing distress.

The present sample primarily consisted of middle to upper socioeconomic status (SES) White women with a high level of education. Replicating this study with a low‐SES sample would provide further understanding of the relations between trauma exposure and maternal mental health (e.g., whether the relation between attachment trauma and postpartum depression would be the same in the higher stress context of poverty). In addition, it is possible that the kind of social support that makes a difference for mothers would differ in a lower income sample (e.g., instrumental support may be more important to those with fewer resources; however, the impact of emotional support may remain that same).

Research on resilience and vulnerability provides valuable insights regarding a mother's ability to “overcome” their trauma and depression (Schachman & Lindsey, 2013). Resilience is the ability to function adaptively both psychologically and physiologically in the face of adversity (Feder, Nestler, & Charney, 2009). A recent study found that resilience served as a buffer against psychiatric symptoms in mothers who experienced childhood trauma (Sexton, Hamilton, McGinnis, Rosenblum, & Muzik, 2015). Incorporating ideas about risk and protective factors into a larger model of maternal mental health could reveal interconnections between postpartum depression, social support, and maternal self‐efficacy that are more compelling than originally anticipated or found by the current model. By accounting for resilience, researchers may be able to explain why some mothers are more affected by their trauma than are others (Easterbrooks, Chaudhuri, Bartlett, & Copeman, 2010).

### Implications for Infant Mental Health

The current findings have implications for professionals working clinically with mothers and their young children. Clinicians should be aware of how the complex range of trauma histories relate to mothers’ psychological well‐being. In addition, this study has implications for the design and implementation of interventions for mothers with postpartum depression. Interventions should not simply focus on providing mothers with more social support; rather, they should facilitate emotional support which could be critical in bolstering self‐efficacy.

The implications of our findings extend to the medical community as well. Research has continually shown that obstetrics and gynecology (OB/GYN) practices fail to assess and treat women's mental health needs, specifically prior depression and trauma exposure (Scholle & Kelleher, 2003). Given that many women turn to their OB/GYN provider as the primary resource for behavioral healthcare (Poleshuck & Woods, 2014; Scholle & Kelleher, 2003), OB/GYN practices present a unique opportunity to provide women access to psychological assessment and treatment as part of their overall perinatal care. It is recommended that OB/GYN providers assess for mental health history and trauma exposure. The earlier a targeted intervention can occur, the less likely that the trauma history will have a long‐lasting impact on their mental health and well‐being. Women with trauma histories are at an increased risk of postpartum depression; thus, it is advised that OB/GYN practices incorporate a screening tool to assess for trauma and prior mental illness into their routine perinatal appointments. By simply asking women about their mental health and trauma exposure, as well as their current symptomology and social support networks, practitioners are setting precedence for high‐quality, wraparound care. In addition, the American Academy of Pediatrics (Workgroup, B. F. P. S., & Committee on Practice and Ambulatory Medicine, 2014) recommended that parents schedule routine appointments with their pediatricians throughout the first 3 years of their child's life. Based on the current study's findings, it is recommended that pediatricians assess for postpartum depression during these visits. Pediatricians tend to have unique access to mothers in the postpartum period, providing an opportunity for screening and discussion regarding postnatal depression.

## CONFLICT OF INTEREST

There were no conflicts of interest for any authors.
